# The Influence of Adolescent Health-related Behaviors on Degenerative Low Back Pain Hospitalizations and Surgeries in Adulthood: A Longitudinal Study

**DOI:** 10.1097/BRS.0000000000005112

**Published:** 2024-08-06

**Authors:** Matias Vaajala, Alisa Teuho, Rasmus Liukkonen, Ville Ponkilainen, Arja Rimpelä, Leena K. Koivusilta, Ville M. Mattila

**Affiliations:** a Faculty of Medicine and Life Sciences, University of Tampere, Tampere, Finland; b Department of Orthopaedics and Traumatology, Tampere University Hospital, Tampere, Finland; c Unit of Health Sciences, Faculty of Social Sciences, Tampere University, Tampere, Finland; d Department of Adolescent Psychiatry, Tampere University Hospital, Tampere, Finland; e Department of Social Research, Faculty of Social Sciences, University of Turku, Turku, Finland

**Keywords:** epidemiology, back pain hospitalization, spine surgery, risk factors

## Abstract

**Study Design.:**

Retrospective longitudinal study.

**Objective.:**

This study aims to investigate the influence of adolescent health-related behaviors (physical activity, high BMI, drunkenness, smoking), self-reported chronic disease, and low socioeconomic status (SES) on the development of low back pain requiring hospitalization or surgery.

**Background.:**

The baseline data were surveys gathered biennially in 1981–1997 (the Adolescent Health and Lifestyle Survey) and individually linked with outcome data, degenerative low back pain hospitalizations, and spine surgeries retrieved from the Care Register for Health Care. A total of 47,724 participants were included. Explanatory variables included physical activity, high BMI, smoking, monthly drunkenness, chronic diseases, and family SES.

**Materials and Methods.:**

A logistic regression model was used to analyze the influence of adolescent health-related behaviors (physical activity, high BMI, drunkenness, smoking), self-reported chronic disease, and low SES on degenerative low back pain hospitalization, lumbar disc herniation (LDH) hospitalization, and/or spine surgery. Covariates were selected using directed acyclic graphs (DAGs).

**Results.:**

A total of 5538 participants had degenerative low back pain hospitalizations, 2104 had LDH hospitalizations, and 913 had spinal surgery over an average of 27-year follow-up. High BMI [adjusted odds ratio (aOR): 1.25, CI: 1.12–1.38], smoking (aOR: 1.53, CI: 1.43–1.62), monthly drunkenness (aOR: 1.17, CI: 1.10–1.26), and chronic diseases (aOR: 1.47, CI: 1.35–1.61) in adolescence increased the odds of hospitalizations during follow-up. In addition, high BMI (aOR: 1.37, CI: 1.09–1.72), smoking (aOR: 1.40, CI: 1.21–1.61), and monthly drunkenness (aOR: 1.19, CI: 1.01–1.39) increased the odds of spine surgeries.

**Conclusions.:**

We found that smoking, high BMI, monthly drunkenness, chronic diseases, and low family SES in adolescence increased the likelihood of degenerative low back pain hospitalizations in adulthood. In addition, high BMI, smoking, and monthly drunkenness in adolescence increased the odds of spinal surgeries.

Back pain is the most common musculoskeletal problem, usually in the lower back.^1^ It is a primary factor behind activity limitations and work absenteeism, imposing a significant medical burden and economic strain.^2^ Consequently, it emerges as one of the prominent worldwide public health challenges.^3,4^ According to the World Health Organization (WHO), low back pain affected 619 million people globally in 2020 and it is estimated that the number of cases will increase to 843 million by 2050.^5^ In Finland, the incidence of back pain-related hospitalizations and surgeries has had a strongly increasing trend.^6–9^

The risk factors of back pain in adulthood are generally well known. Factors such as sedentary lifestyles, poor posture, obesity, occupational hazards, and mental health problems contribute substantially to the prevalence of back pain-related hospitalizations.^10,11^ In addition, jobs requiring heavy lifting, prolonged sitting, or repetitive motions increase the risk of developing chronic back issues that may necessitate surgical intervention.^11,12^ However, less is known about the risk factors in childhood and adolescence that predict back pain problems in adulthood, even though these are highly related from adolescence to adulthood.^13^ The relation is found to be highest in smoking, and alcohol use.^13^ Adolescence marks a critical period of physical and psychological development, when adopting several health-related behaviors can have long-lasting effects on musculoskeletal health.^14,15^ In a systematic review of the risk factors for episodes of back pain in younger adults, no consistent associations were found for lifestyle factors such as physical activity or BMI.^16^ This systematic review found, however, that a previous episode of back pain was a consistent risk factor for a new episode of back pain across several studies included in the systematic review.^16^ A British longitudinal study in 2018 investigated the childhood risk factors for adulthood back pain and found that abdominal pain, poorest care in childhood, and poorer maternal health increased the risk for back pain.^17^ In addition, increased activity in sports can be a predisposing factor, for example, lumbar disc herniation (LDH) and stress fractures of the spine.^18^ Spondylolysis occurs in highly active individuals due to repetitive stress and strain on the lower back, leading to a stress fracture in the vertebra.^19^ Over time, this can cause instability, pain, and potential nerve compression.^20^

The literature assessing the effects of adolescent risk factors on the development of low back pain hospitalizations or spine surgeries in adulthood is currently insufficient and contradictory. Hence, this study aims to investigate the influence of physical activity, high BMI, alcohol use, smoking, chronic disease, and family SES in adolescence on the development of hospitalization or surgery requiring low back pain in a large cohort of Finnish adolescents with an average 27-year follow-up.

## MATERIALS AND METHODS

### Study Design

In this longitudinal study, the survey data from the Adolescent Health and Lifestyle Survey (AHLS) were individually linked with sociodemographic data from Statistics Finland and with outcome data retrieved from the Care Register for Health Care (formerly the Hospital Discharge Register).^21^ The endpoint of the follow-up for each participant was the first occurrence of degenerative low back pain hospitalization, LDH hospitalization, or surgery, or the termination of the follow-up on December 31, 2018.

### Baseline Data

The baseline data were sourced from AHLS.^22^ Commencing in 1977, surveys were mailed biennially to all Finns aged 14, 16, or 18, born on certain days in June, July, or August. The surveys took place between February and March, with individual follow-ups commencing from the conclusion of each survey on April 30 of the survey year. Samples were drawn from the Population Register Centre. Two reinquiries were sent to nonresponders. The study utilized data collected between 1981 and 1997. If the responders had answered more than one survey, the answer from his/her first survey was used. The overall response rate was 77.8%.

### Outcome Variable

Three outcome variables were analyzed: (1) degenerative low back pain hospitalization, (2) LDH hospitalization, and (3) spine surgery. We included only the first degenerative low back pain hospitalization diagnosis, LDH diagnosis, and spine surgery code in the models. Outcome variables were obtained from the Care Register for Health Care, which includes information on participants discharged from inpatient care, day surgeries, and specialized outpatient care. The coverage and quality of the Care Register for Health Care is good.^23^ ICD-9 and ICD10 (International Classification of Diseases 9th 10th revision) diagnoses, were used to identify specific degenerative low back-pain hospitalizations starting from the year 1998, as the quality of the register has higher quality since then. The NOMESCO (Nordic Medico-Statistical Committee) operation codes, also found in the Care Register for Health Care, were used to identify participants who underwent degenerative spine surgery. We analyzed LDH hospitalizations separately from all degenerative low back pain hospitalizations because the majority of degenerative low back pain–related diagnoses are often first diagnosed as unspecific back pain. Spine contusions, spine fractures, and spine fracture surgeries are not included in this study because these are more likely caused by high-energy traumas rather than degenerative processes due to lifestyle and health status of the spine.^24^ The specific ICD codes and NOMESCO operation codes included in this study are shown in Supplemental Table 1 (Supplemental Digital Content 1, http://links.lww.com/BRS/C491).

**Figure 1:**
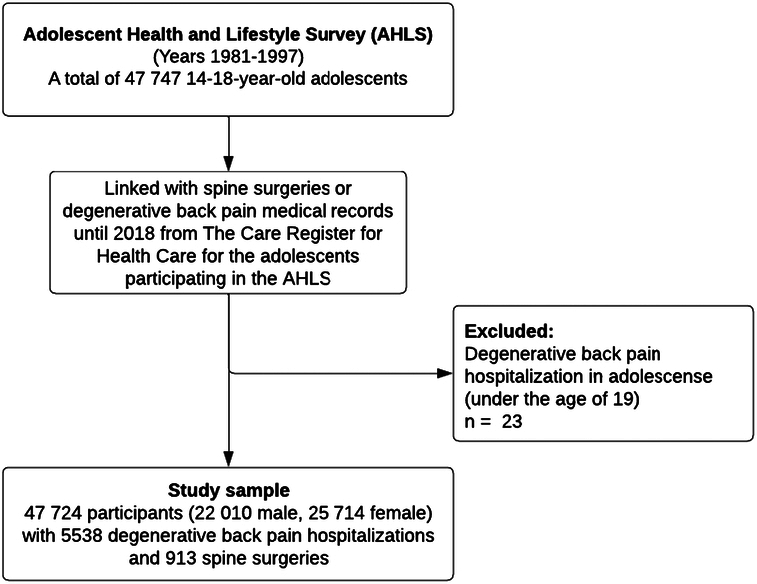
Flowchart depicting the formation of the study sample. Data from the Adolescent Health and Lifestyle Survey were linked with the data on spine surgeries or degenerative low back pain medical records in the Care Register for Health Care.

Adolescents with degenerative low back pain hospitalizations under age 19 were excluded from the study (n=23). A total of 47,724 participants were included in the final study sample. Of these, 22,010 were male and 25,714 females. The flowchart for forming the final study sample is shown in Figure 1.

### Explanatory Variables

Variables describing health behaviors and chronic disease were obtained from the AHLS and family SES from the National Registries of Statistics Finland. A summary of the variables used in the analyses and original variables are shown in Table 1.

**TABLE 1:** Description of Explanatory Variables Used in the Analyses

Variable and its source	Original variable and formation of the variable used in the analyses	Values
Family SES, Statistics Finland	Occupation-based socioeconomic status of the respondent’s mother and father from the National Registries of Statistics Finland. The registry data on socioeconomic circumstances had been obtained from national censuses conducted every fifth year until 1995 and from on-line registry data on a yearly basis from 2000 onwards. Classification of Statistics Finland^25^	Both parents’ unknown=0 Both parents upper white-collar=1 Either one upper white-collar=2 Either one lower white-collar=3 Either one blue-collar=4
Daily use of tobacco, AHLS	Combining questions on tobacco experimentation and frequency of tobacco use	No=1 Yes=2
Monthly drunkenness, AHLS	Question on frequency of alcohol use and drunkenness	No=1 (abstinence or only occasional drinking) Yes=2 (drunk once or more often a month)
Frequency of physical activity, AHLS	Combining questions on frequency of participation in physical exercise in sports clubs and frequency of leisure time physical activity	Low=1 (once a week or less) Medium=2 (2–3 times a week or less) High=3 (4 or more times a week)
Self-reported chronic diseases and disabilities AHLS	Question on long-term disease or disability that disturbs your everyday life	No=1 Yes=2
Overweight AHLS	BMI calculated from self-reported height (cm) and weight (kg)	No=1 (normal weight) Yes=2 (high BMI according to Cole’s criteria)^26^

AHLS indicates Adolescent Health and Lifestyle Survey; BMI, body mass index; SES, socioeconomic status.

### Statistical Methods

Continuous variables were presented as means with SD. Categorical variables were presented as absolute numbers and rates. A logistic regression model was used to risk factors for the outcomes: degenerative low back pain hospitalizations, LDH hospitalizations, and spine surgeries. The explanatory variables were adolescent health and health behavior variables: physical activity, high BMI, smoking, monthly drunkenness, chronic diseases, and family SES. In the models for family SES, adolescents with a missing SES of both parents were excluded from the model. The gender-stratified models were also created, and the results are shown in supplementary files (Supplemental Tables 2, Supplemental Digital Content 2, http://links.lww.com/BRS/C492 and 3, Supplemental Digital Content 3, http://links.lww.com/BRS/C493). Adjusted odds ratios (aOR) with 95% CIs for explanatory variables were compared between groups.

In logistic regression models, adjustments were made by choosing the variables for a multivariable model using directed acyclic graphs (DAGs). The variables included in the DAGs were chosen based on known risk factors and hypothesized causal pathways.^5,27^ The models were created using the free online software DAGitty (dagitty.net) (Supplemental Figs. 1–5, Supplemental Digital Content 4, http://links.lww.com/BRS/C494, Supplemental Digital Content 5, http://links.lww.com/BRS/C495, Supplemental Digital Content 6, http://links.lww.com/BRS/C496, Supplemental Digital Content 7, http://links.lww.com/BRS/C497, Supplemental Digital Content 8, http://links.lww.com/BRS/C498).^28^ DAGitty automatically suggests possible adjustment variable sets that can influence the main outcome. DAGitty determines the minimal adjustment set needed to block all noncausal paths, ensuring no node in the set is a descendant of the explanatory and that all backdoor paths are blocked when conditioning on this set. The tool uses algorithms from graph theory and causal inference to automate this process and provides real-time feedback on the sufficiency of specified adjustments, aiding researchers in making valid causal inferences.^29^ The DAGs for each explanatory variable are shown as supplementary figures (Supplemental Figs. 1–6, Supplemental Digital Content 4, http://links.lww.com/BRS/C494, Supplemental Digital Content 5, http://links.lww.com/BRS/C495, Supplemental Digital Content 6, http://links.lww.com/BRS/C496, Supplemental Digital Content 7, http://links.lww.com/BRS/C497, Supplemental Digital Content 8, http://links.lww.com/BRS/C498), Supplemental Digital Content 9, http://links.lww.com/BRS/C499). Family SES, physical activity, and smoking in adolescence were used as adjusting variables in the analyses.

Variance inflation factor (VIF) scores were calculated to assess the potential for regression model instability.^30^ Statistical analyses were performed with R version 4.0.5 (R Foundation for Statistical Computing, Vienna, Austria).^31^ The results of this study are reported according to the STROBE (Strengthening the Reporting of Observational Studies in Epidemiology) guidelines.^32^

## RESULTS

Among the participants, the age at baseline was distributed nearly equally between the ages 14, 16, and 18 years. Descriptive statistics for the adolescents are shown in Table 2.

**TABLE 2:** Descriptive Statistics on Variables Used in the Study by Sex

	All	Male	Female
	47,727	22,010	25,714
Total number	n (%)	n (%)	n (%)
Background information			
Age during the survey			
14 y	15,880 (33.3)	7533 (34.2)	8347 (32.5)
16 y	15,885 (33.3)	7320 (33.3)	8565 (33.3)
18 y	15,982 (33.5)	7167 (32.6)	8815 (34.3)
Age at the end of the follow-up (y) (mean; SD)	42.7 (4.3)	42.7 (4.3)	42.7 (4.3)
Family SES at age 15			
Both upper white-collar	7775 (16.3)	3677 (16.7)	5776 (22.5)
Either one upper white-collar	11,899 (24.9)	5456 (24.8)	1319 (5.1)
Either one lower white-collar	15,184 (31.8)	7093 (32.2)	8091 (31.5)
Either on blue-collar	10,423 (21.8	4647 (21.1)	4098 (16.0)
Both unknown	2466 (5.2)	1147 (5.2)	6443 (25.1)
Explanatory variables			
Smoking in adolescence	11,564 (24.2)	5690 (25.9)	5874 (22.8)
Drunk once or more a month	9048 (19.0)	4892 (22.2)	4156 (16.2)
Physical activity in adolescence			
High	11,315 (23.7)	5588 (25.3)	5727 (22.3)
Medium	13,467 (28.2)	6324 (28.7)	7143 (27.8)
Low	22,280 (46.7)	9681 (44.0)	12,599 (49.0)
Unknown	662 (1.4)	417 (1.9)	245 (1.0)
High BMI in adolescence	4839 (10.1)	2838 (12.9)	2001 (7.8)
Chronic diseases in adolescence	4222 (8.8)	1819 (8.2)	2403 (9.3)
Outcome variables in adulthood			
Low back pain hospitalizations	5538 (11.6)	2524 (11.5)	3014 (11.7)
LDH hospitalizations	2104 (4.4)	1084 (4.9)	1020 (4.0)
Spine surgeries	913 (1.9)	490 (2.2)	423 (1.6)

BMI indicates body mass index; LDH, lumbar disc herniation; SES, socioeconomic status.

The most common first degenerative back pain hospitalization diagnoses were low back pain (*M*=54.5) (n=1781, 32.2%), disc disorders with radiculopathy (*M*=51.1) (n=1338, 24.2%), and unspecified dorsalgia (*M*=54.9) (n=669, 12.1%). The mean age at the time of the first degenerative back pain hospitalization diagnosis was 34.1 years (SD=7.4). The most common first LDH hospitalization diagnosis was disc disorders with radiculopathy (*M*=51.1) (n=2029, 96.4%). The mean age at the time of the first LDH hospitalization diagnosis was 33.6 years (SD=7.0). The most common spine surgeries were excision of lumbar intervertebral disc displacement (ABC16) (n=487, 53.3%), open discectomy of lumbar spine (ABC26) (n=279, 30.6%), and decompression of lumbar nerve roots ABC36 (n=92, 10.1%). The mean age at the time of spine surgeries was 34.7 years (SD=6.6).

The adjustments for the logistic regression models were chosen based on the DAG method (Supplemental Figs. 1–6, Supplemental Digital Content 4, http://links.lww.com/BRS/C494, Supplemental Digital Content 5, http://links.lww.com/BRS/C495, Supplemental Digital Content 6, http://links.lww.com/BRS/C496, Supplemental Digital Content 7, http://links.lww.com/BRS/C497, Supplemental Digital Content 8, http://links.lww.com/BRS/C498, Supplemental Digital Content 9, http://links.lww.com/BRS/C499). The models with physical activity, smoking, monthly drunkenness, and chronic diseases as an exposure variable were adjusted by the age at the end of the follow-up and family SES in adolescence. The model with BMI as an exposure variable was adjusted by the age at the end of the follow-up, physical activity, and family SES in adolescence. The model with Family SES as an exposure variable was adjusted by the age at the end of the follow-up, and smoking status in adolescence.

In the logistic regression analysis, adolescents with high BMI had higher odds for all degenerative low back pain hospitalizations in adulthood when compared with those with normal weight (all aOR: 1.25, CI: 1.12–1.38; males aOR: 1.17, CI: 1.01–1.34; females aOR: 1.36, CI: 1.17–1.58) (Table 3). Correspondingly, in comparison to nonsmoking adolescents, smoking increased the odds for all adolescents (aOR: 1.53, CI: 1.43–1.62), males (aOR: 1.53, CI: 1.41–1.67), and females (aOR: 1.53, CI: 1.41–1.67), when compared with nonsmoking adolescents. Recurrent drinking or drunkenness increased the odds for all adolescents (aOR: 1.17, CI: 1.10–1.26), males (aOR 1.16, CI 1.05–1.27), and females (aOR 1.20, CI 1.09–1.32), when compared with adolescents without recurrent drinking. In addition, adolescents with chronic diseases had increased odds of degenerative low back pain hospitalizations in adulthood for all adolescents (aOR: 1.47, CI: 1.35–1.61), males (aOR: 1.40, CI: 1.22–1.60), and females (aOR: 1.53, CI: 1.36–1.72). Also, adolescents with lower family SES had higher odds of degenerative back pain hospitalizations (aOR: 1.18, CI: 1.12–1.23). We found no evidence of a difference in physical activity and the likelihood of degenerative low back pain hospitalizations (Table 3).

**TABLE 3:** Adjusted Odds Ratios (aOR) With 95% CI for the Primary Outcomes: Degenerative Low Back Pain Hospitalizations, Lumbar Disc Herniation (LDH) Hospitalization, and Spine Surgeries

	Degenerative back pain hospitalization		LDH hospitalizations		Spine surgery	
	aOR	CI	aOR	CI	aOR	CI
Physical activity^a^						
Low	1.00		1.00		1.00	
Medium	0.99	0.93–1.06	0.96	0.86–1.07	1.00	0.86–1.17
High	1.04	1.00–1.07	1.02	0.96–1.08	1.02	0.94–1.11
BMI^b^						
Normal BMI	1.00		1.00		1.00	
High BMI	**1.25**	**1.12–1.38**	**1.47**	**1.26–1.71**	**1.37**	**1.09–1.72**
Tobacco use^a^						
No smoking	1.00		1.00		1.00	
Smoking	**1.53**	**1.43–1.62**	**1.52**	**1.38–1.67**	**1.40**	**1.21–1.61**
Monthly drunkenness^a^						
Abstinence or occasional	1.00		1.00		1.00	
Drunk once or more a month	**1.17**	**1.10–1.26**	**1.18**	**1.06–1.31**	**1.19**	**1.01–1.39**
Chronic disease^a^						
No	1.00		1.00		1.00	
Yes	**1.47**	**1.35–1.61**	**1.30**	**1.12–1.49**	1.02	0.81–1.27
Family socioeconomic status^c^						
Both parents upper white-collar	1.00		1.00		1.00	
Either one upper white-collar	**1.14**	**1.03–1.26**	1.09	0.94–1.26	1.05	0.84–1.31
Either one lower white-collar	**1.09**	**1.04–1.14**	1.06	0.99–1.14	1.03	0.93–1.14
Either one blue-collar	**1.18**	**1.12–1.23**	**1.15**	**1.07–1.24**	1.03	0.92–1.15

Statistically significant findings are marked in bold text.

^a^ Adjusted by the age at the end of the follow-up and family socioeconomic status in adolescence.

^b^ Adjusted by the age at the end of the follow-up, physical activity, and family socioeconomic status in adolescence.

^c^ Adjusted by the age at the end of the follow-up, and smoking status in adolescence.

BMI indicates body mass index.

In the logistic regression model for LDH hospitalizations, high BMI for all adolescents (aOR: 1.47, CI: 1.26–1.71) was found to increase the odds for LDH hospitalizations in adulthood, when compared with normal-weight adolescents. Smoking increased the odds of LDH hospitalizations in adulthood for all adolescents (aOR: 1.52, CI: 1.38–1.67), males (aOR: 1.48, CI: 1.29–1.68), and females (aOR: 1.54, CI: 1.34–1.76) when compared with nonsmoking adolescents. Also, chronic diseases in adolescence increased the odds of LDH hospitalizations in adulthood for all adolescents (aOR: 1.30, CI: 1.12–1.49), males (aOR: 1.29, CI: 1.05–1.58), and females (aOR: 1.32, CI: 1.08–1.59). Also, adolescents with the lowest family SES had increased odds of LDH hospitalizations in adulthood (aOR: 1.15, CI: 1.07–1.24). We found no evidence of a difference in the high physical activity and the likelihood of degenerative low back pain hospitalizations (Table 3).

In the model for spine surgeries, high BMI for all adolescents increased the odds for spine surgeries in adulthood (aOR: 1.37, CI: 1.09–1.72), when compared with normal-weight adolescents. Smoking increased the odds of spine surgeries in adulthood for all adolescents (aOR: 1.40, CI: 1.21–1.61), males (aOR: 1.32, CI: 1.09–1.61), and females (aOR: 1.45, CI: 1.17–1.78), when compared with nonsmokers. Monthly drunkenness increased the odds of spine surgeries in adulthood for all adolescents when compared with adolescents without recurrent drinking (aOR: 1.19, CI: 1.01–1.39). We found no evidence of a difference in the high physical activity and the likelihood of spine surgeries (Table 3).

## DISCUSSION

Our main finding was that smoking, high BMI, monthly drunkenness, chronic diseases, and low SES increased the likelihood of degenerative low back pain hospitalizations in adulthood. In addition, high BMI, smoking, and monthly drunkenness were linked to a higher likelihood of future spinal surgeries. High BMI and smoking also increased the likelihood of LDH hospitalizations. However, the frequency of physical activity in adolescence was not associated with degenerative low back pain hospitalizations, spinal surgeries, or LDH hospitalizations in adulthood.

For adolescent smoking and alcohol use, etiological factors may explain part of the findings. Nicotine in cigarettes can decrease blood flow to the spinal discs, leading to decreased nutrient supply and impaired healing capacity.^33^ In other studies, too, health-compromising behaviors have been positively associated with LDH hospitalizations in both sexes. In addition, smoking has been linked to reduced bone density and increased risk of osteoporosis, which can contribute to spinal problems such as fractures and degenerative disc disease.^34–36^ Alcohol use can weaken bones and muscles,^37,38^ making individuals more susceptible to spinal injuries and chronic pain conditions. Adolescents who engage in health-compromising behaviors also report a higher number of mental health problems like stress, depression, or anxiety, which can exacerbate or contribute to chronic pain conditions like back pain.^39,40^ Also, the consequences of smoking and heavy alcohol consumption during adolescence may not manifest until later in life. Chronic exposure to these substances during critical periods of growth and development can have lasting effects on the body’s structure and function, predisposing individuals to degenerative low back pain in adulthood.^41^ Further research is needed to elucidate the specific mechanisms underlying the relationship between early use of tobacco and alcohol, and the subsequent health of the back and to develop targeted interventions aimed at reducing the burden of back pain in at-risk populations.

Unexpectedly, participating in sports in adolescence was not associated with degenerative low back pain hospitalizations. In previous literature, it has been speculated that the increased stress that certain sports activities impose on the back leads to nullification or even exacerbation of the protective effects of exercise.^42,43^ Many sports, especially those involving repetitive or high-impact movements such as running, jumping, or weightlifting, can place significant stress on the spine.^42,43^ Activities like football, gymnastics, or heavy weightlifting can subject the back to forces that may exceed its physiological capacity, leading to microtrauma, disc degeneration, or even acute injuries like herniated discs or vertebral fractures.^42,43^ Our study could not confirm these speculations—participation in sports did not increase the likelihood of spinal surgeries in our sample.

According to our results, a high BMI was positively associated with degenerative low back pain hospitalizations, including spine surgeries and LDH hospitalizations. A systematic review of the risk factors for episodic back pain in young adults did not find consistent support for lifestyle factors such as physical activity level or high BMI, even though these were highlighted as important risk factors in some earlier studies.^16^ However, this systematic review did not have any longitudinal studies included. Obesity exacerbates back pain by placing extra mechanical stress on the spine, altering its natural alignment, and promoting degenerative changes like osteoarthritis.^44,45^ Obesity associated with sedentary lifestyle weakens spinal-supporting muscles, while adipose tissue produces inflammatory cytokines, aggravating inflammation in the spine, too.^46^

Interestingly, chronic diseases in adolescence had an impact on the likelihood of degenerative low back pain hospitalizations. On the contrary, the odds of spinal surgeries were not higher. Due to numerous limitations in the data for the chronic diseases variable, the exact reasons for these findings remain unknown. In previous literature, long-term medication use, such as corticosteroids for asthma, can weaken bones, increasing vulnerability to spinal issues like osteoporosis, even among adolescents.^47,48^ In addition, psychosocial stressors related to managing chronic diseases in everyday life can lead to mental health problems, contributing to back pain over time.^49^ Also, as the odds for spinal surgeries were not higher, one possibility is that hospitalizations may be more related to the overall complexity and comorbidity burden of patients with chronic diseases rather than solely due to spine disorders. However, as there is no differentiation between the chronic diseases, conclusions on this association cannot be, and this topic should be better studies using more precise data sets.

A strength of this study lies in its long follow-up period, 27 years on average, which could show how adolescent risk factors can influence adult low back pain hospitalizations even in mid-adulthood. Our nationwide sample was large representing Finnish adolescents. The study does possess limitations. All health behavior and chronic disease variables were self-reported, which may cause inaccuracy and bias in results. In survey-based studies, recall bias is a common limitation, as participants might not remember past events accurately, often underreporting or overreporting information. Survey bias, on the other hand, arises when the survey design, question phrasing, or respondents’ willingness to answer truthfully influences the results. For example, younger adolescents reporting smoking or alcohol use may be biased because they have been afraid that parents see their answers. On the other hand, the reliability and validity of self-reported smoking status are found to be relatively good among young adults.^50^ Moreover, we do not know how health and health behaviors have changed over time. However, these (especially smoking) are known to be clearly related from adolescence to adulthood.^13^ In addition, another weakness is the lack of granularity in the data about chronic diseases, as we have no information on the types of chronic diseases, and therefore, they are treated as equal, which limits the conclusions that can be drawn from our study.

## CONCLUSIONS

The main finding of this study was that smoking, high BMI, monthly drunkenness, chronic diseases, and low family SES in adolescence increased the likelihood of future degenerative low back pain hospitalizations in a follow-up of 27 years. In addition, high BMI, smoking, and monthly drunkenness increased the likelihood of future spinal surgeries. However, participating in sports in adolescence was not associated with degenerative low back pain hospitalizations, spine surgeries, or LDH hospitalizations in adulthood. These associations should be further studied to determine ways to prevent low back pain hospitalizations and spine surgery in adulthood as well as to potentiate early interventions.

Key Points

- Smoking, high BMI, monthly drunkenness, chronic diseases, and low family SES in adolescence increased the likelihood of degenerative low back pain hospitalizations in adulthood.
- High BMI, smoking, and monthly drunkenness increased the odds of spinal surgeries.
- Efforts aimed at these can be effective in preventing hospital treatments measured in the context of back pain-related hospitalizations.

## Supplementary Material
