# Supplementary material for: The Influence of Adolescent Health-related Behaviors on Degenerative Low Back Pain Hospitalizations and Surgeries in Adulthood: A Longitudinal Study
Source: Spine (Phila Pa 1976). 2024 Aug 6;49(24):1750–7. doi: 10.1097/BRS.0000000000005112 (PMC11581437; doi:10.1097/BRS.0000000000005112)
Supplement: Supplementary file 1 [file brs-49-1750-s001.docx]

**Supplementary Table 1**: The classification of hospitalizations due to degenerative low back pain and spine surgeries according to diagnostic and procedure codes.

| **Degenerative low back pain** | **Spine surgeries** |
| --- | --- |
| **ICD-codes (ICD-9 codes*)** | **NOMESCO operation codes** |
| **Non-specific back pain** | **Disc surgery** |
| **M53.8** Other specified dorsopathies | **ABC16** Excision of lumbar intervertebral disc displacement |
| **M53.9** Dorsopathy, unspecified | **ABC26** Open discectomy of lumbar spine |
| **M54.0 / 723.6*** Panniculitis of back | **ABC36** Decompression of lumbar nerve roots |
| **M54.3** Ischias | **ABC56** Decompression of lumbar spinal canal and nerve roots |
| **M54.4 / 724.2*** Lumbago with or without sciatica | **NAG53** Interbody fusion of thoraco-lumbar spine with external fixation |
| **M54.5** Low back pain | **NAG57** Interbody fusion of spine with external fixation |
| **M54.6 / 724.1*** Pain in thoracic spine | **NAG61** Posterior fusion of lumbar spine without fixation |
| **M54.8** Other dorsalgia | **NAG63** Interlaminary fusion of thoraco-lumbar spine without fixation |
| **M54.9** Dorsalgia, unspecified | **NAG65** Anterior and posterior fusion of lumbar spine |
| **724.4*** Back pain with radiation | **NAG66** Interlaminary fusion of lumbo-sacral spine without fixation |
| **724.5*** Unspecified back pain | **NAG99** Other excision, reconstruction, or fusion |
| **Lumbar disc herniation** |  |
| **M51.0 / 722.7*** Intervertebral disc disorders with myelopathy |  |
| **M51.1** Disc disorders with radiculopathy |  |
| **M51.9** Unspecified intervertebral disc disorder |  |
| **722.1*** Lumbar disc displacement |  |
| **722.51* / 722.52*** Degeneration of thoracic or lumbar intervertebral disc |  |
| **Spinal stenosis** |  |
| **M48.0 / 723.0* / 724.0*** Spinal stenosis |  |
| **M47.2 / 721.0-721.4*** Spondylosis with or without radiculopathy |  |

* ICD-9 codes
