# Supplementary material for: The Influence of Adolescent Health-related Behaviors on Degenerative Low Back Pain Hospitalizations and Surgeries in Adulthood: A Longitudinal Study
Source: Spine (Phila Pa 1976). 2024 Aug 6;49(24):1750–7. doi: 10.1097/BRS.0000000000005112 (PMC11581437; doi:10.1097/BRS.0000000000005112)
Supplement: Supplementary file 2 [file brs-49-1750-s002.docx]

**Supplementary table 2**. Gender-stratified for males. Adjusted odds ratios (aOR) with 95% confidence intervals (CI) for the primary outcomes: degenerative low back pain hospitalizations, lumbar disc herniation (LDH) hospitalizations, and spine surgeries. * Statistically significant findings are marked in bold text.

|  | Degenerative back pain hospitalization | | LDH hospitalization | |  | Spine surgery | |  |
| --- | --- | --- | --- | --- | --- | --- | --- | --- |
|  | aOR (β*) | CI | aOR (β*) | CI | | aOR (β*) | CI | |
| Physical activity^a^ |  |  |  |  | |  |  | |
| low | 1.00 |  | 1.00 |  | | 1.00 |  | |
| medium | 1.03 | 0.97-1.07 | 1.02 | 0.88-1.18 | | 1.00 | 0.80-1.23 | |
| high | **1.06** | **1.01-1.12** | 1.03 | 0.95-1.11 | | 1.05 | 0.94-1.17 | |
| BMI^b^ |  |  |  |  | |  |  | |
| normal BMI | 1.00 |  | 1.00 |  | | 1.00 |  | |
| high BMI | **1.17** | **1.01-1.34** | **1.34** | **1.09-1.63** | | 1.24 | 0.91-1.65 | |
| Tobacco^a^ |  |  |  |  | |  |  | |
| no smoking | 1.00 |  | 1.00 |  | | 1.00 |  | |
| smoking | **1.53** | **1.41-1.67** | **1.48** | **1.29-1.68** | | **1.32** | **1.09-1.61** | |
| Monthly drunkenness^a^ |  |  |  |  | |  |  | |
| abstinence or occasional | 1.00 |  | 1.00 |  | | 1.00 |  | |
| drunk once or more a month | **1.16** | **1.05-1.27** | 1.12 | 0.94-1.31 | | 1.18 | 0.91-1.50 | |
| Chronic diseases^a^ |  |  |  |  | |  |  | |
| no chronic diseases | 1.00 |  | 1.00 |  | | 1.00 |  | |
| one or more | **1.40** | **1.22-1.60** | **1.29** | **1.05-1.58** | | 1.10 | 0.80-1.49 | |
| Family socioeconomic status^c^ |  |  |  |  | |  |  | |
| Both parents upper white-collar | 1.00 |  | 1.00 |  | | 1.00 |  | |
| Either one upper white-collar | 1.08 | 0.94-1.25 | 1.07 | 0.87-1.31 | | 1.01 | 0.76-1.36 | |
| Either one lower white-collar | 1.07 | 1.00-1.15 | 1.04 | 0.95-1.15 | | 0.96 | 0.84-1.11 | |
| Either one blue-collar | **1.18** | **1.10-1.26** | 1.18 | 1.07-1.24 | | 1.04 | 0.89-1.19 | |

^a^ Adjusted by the age at the end of the follow-up and family socioeconomic status in adolescence

^b^ Adjusted by the age at the end of the follow-up, physical activity, and family socioeconomic status in adolescence

^c^ Adjusted by the age at the end of the follow-up, and smoking status in adolescence
