# Supplementary material for: The Influence of Adolescent Health-related Behaviors on Degenerative Low Back Pain Hospitalizations and Surgeries in Adulthood: A Longitudinal Study
Source: Spine (Phila Pa 1976). 2024 Aug 6;49(24):1750–7. doi: 10.1097/BRS.0000000000005112 (PMC11581437; doi:10.1097/BRS.0000000000005112)
Supplement: Supplementary file 3 [file brs-49-1750-s003.docx]

**Supplementary table 3**.Gender-stratified analysis for females. Adjusted odds ratios (aOR) with 95% confidence intervals (CI) for the primary outcomes: degenerative low back pain hospitalizations, lumbar disc herniation (LDH) hospitalizations, and spine surgeries. * Statistically significant findings are marked in bold text.

|  | Degenerative back pain hospitalization | | LDH hospitalization | |  | Spine surgery | |  |  |
| --- | --- | --- | --- | --- | --- | --- | --- | --- | --- |
|  | aOR | CI | aOR | CI | | aOR | CI | |  |
| Physical activity^a^ |  |  |  |  | |  |  | |  |
| low | 1.00 |  | 1.00 |  | | 1.00 |  | |  |
| medium | 0.96 | 0.88-1.06 | 0.89 | 0.76-1.03 | | 1.00 | 0.79-1.24 | |  |
| high | 1.02 | 0.97-1.07 | 0.99 | 0.92-1.07 | | 0.96 | 0.85-1.09 | |  |
| BMI^b^ |  |  |  |  | |  |  | |  |
| normal BMI | 1.00 |  | 1.00 |  | | 1.00 |  | |  |
| high BMI | **1.36** | **1.17-1.58** | **1.55** | **1.21-1.96** | | 1.44 | 0.98-2.05 | |  |
| Tobacco^a^ |  |  |  |  | |  |  | |  |
| no smoking | 1.00 |  | 1.00 |  | | 1.00 |  | |  |
| smoking | **1.53** | **1.41-1.67** | **1.54** | **1.34-1.76** | | **1.45** | **1.17-1.78** | |  |
| Monthly drunkenness^a^ |  |  |  |  | |  |  | |  |
| abstinence or occasional | 1.00 |  | 1.00 |  | | 1.00 |  | |  |
| drunk once or more a month | **1.20** | **1.09-1.32** | **1.30** | **1.12-1.49** | | 1.18 | 0.91-1.50 | |  |
| Chronic diseases^a^ |  |  |  |  | |  |  | |  |
| no chronic diseases | 1.00 |  | 1.00 |  | | 1.00 |  | |  |
| one or more | **1.53** | **1.36-1.72** | **1.32** | **1.08-1.59** | | 0.95 | 0.67-1.31 | |  |
| Family socioeconomic status^c^ |  |  |  |  | |  |  | |  |
| Both parents upper white-collar | 1.00 |  | 1.00 |  | | 1.00 |  | |  |
| Either one upper white-collar | **1.19** | **1.04-1.36** | 1.11 | 0.89-1.39 | | 1.10 | 0.79-1.55 | |  |
| Either one lower white-collar | **1.10** | **1.04-1.18** | 1.08 | 0.97-1.20 | | 1.11 | 0.95-1.31 | |  |
| Either one blue-collar | **1.18** | **1.10-1.25** | 1.13 | 1.02-1.26 | | 1.05 | 0.87-1.23 | |  |

^a^ Adjusted by the age at the end of the follow-up and family socioeconomic status in adolescence

^b^ Adjusted by the age at the end of the follow-up, physical activity, and family socioeconomic status in adolescence

^c^ Adjusted by the age at the end of the follow-up, and smoking status in adolescence
