# Supplementary material for: The Influence of Adolescent Health-related Behaviors on Degenerative Low Back Pain Hospitalizations and Surgeries in Adulthood: A Longitudinal Study
Source: Spine (Phila Pa 1976). 2024 Aug 6;49(24):1750–7. doi: 10.1097/BRS.0000000000005112 (PMC11581437; doi:10.1097/BRS.0000000000005112)
Supplement: Supplementary file 4 [file brs-49-1750-s004.pdf]

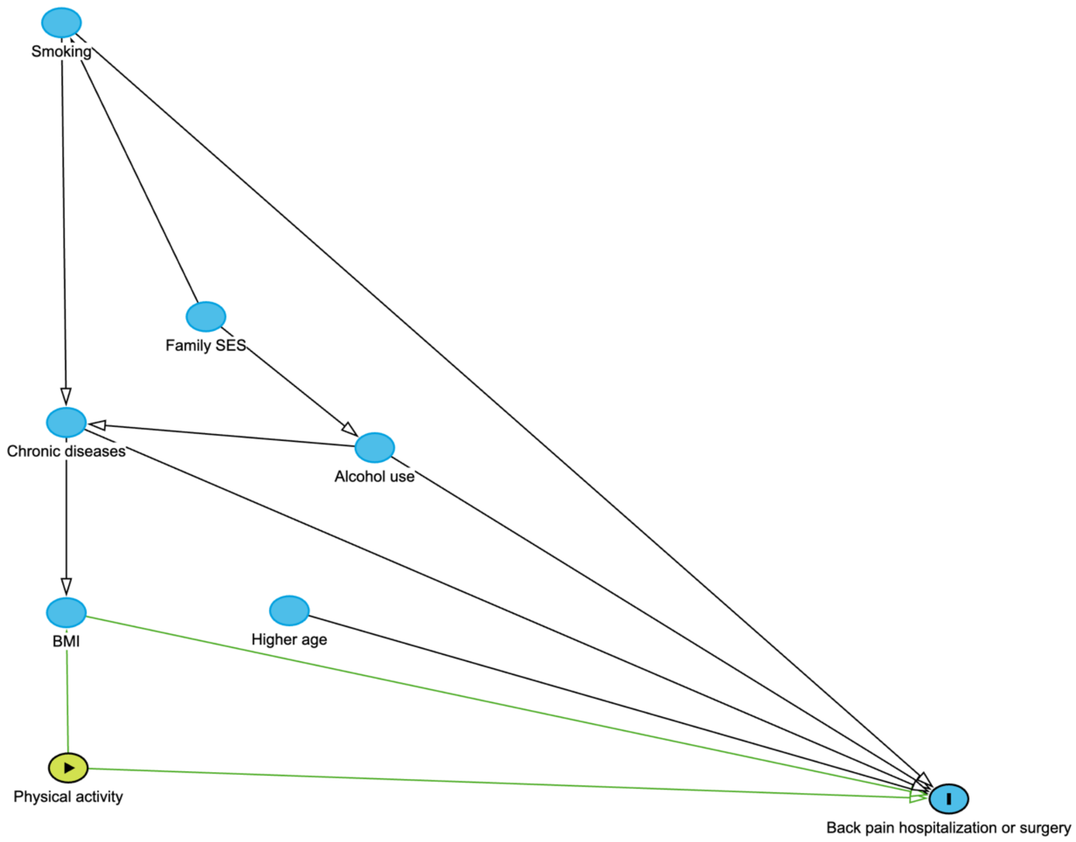

Supplementary Figure 1. DAG: Physical activity and the risk for degenerative low back pain hospitalizations or spine surgeries.
